# Myelomastocytic Blast Cell Crisis in Resistant Tyrosine Kinase Inhibitor Chronic Myelogenous Leukemia: Case Report and Review of Literature

**DOI:** 10.7759/cureus.4703

**Published:** 2019-05-21

**Authors:** Humberto Martinez-Cordero, Bonell Patiño-Escobar, Leonardo J Enciso, Diana M Otero, Paola Spirko

**Affiliations:** 1 Hematology, National Cancer Institute, Bogotá, COL

**Keywords:** mast cell leukemia, chronic myeloid leukemia, myeloid blast crisis, leukemia, hypercalcemia, central nervous system (cns) complications, mast cells, intracranial bleed

## Abstract

We present the clinical case of a 29-year-old male with a diagnosis of chronic myeloid leukemia (CML) in high-risk chronic phase since February 2010. He started treatment with imatinib at a dose of 400 mg obtaining a hematologic response early but without reaching a cytogenetic response in month 18. Then, dasatinib was prescribed. The BCR-ABL transcription level of 58% was documented. It was decided to start treatment with nilotinib but in March 2017 we diagnosed a progression to blast crisis (BC) of myeloid origin with a bone marrow study that documented 72% of blasts with normal karyotype, also very striking, the concomitant skin infiltration, bone lesions of lytic type and hypercalcemia that required the use of zoledronic acid as an emergency. At the end of chemotherapy induction with 7 + 3 (seven days of cytarabine and three days of idarubicin) chemotherapy associated with bosutinib for 14 days and after several infectious complications, we documented a percentage of blasts by flow cytometry of 29% in the bone marrow and the existence of 46% of cells with basophilic characteristics versus mast cells. A basophilic transformation was suspected versus aggressive systemic mastocytosis with a clonal, nonmastocytic hematological disorder. Levels of serum tryptase and mutation D816V C KIT were requested, which were not possible to perform. Treatment with CLAG-M was proposed, however, the patient died early with hyperleukocytosis and severe thrombocytopenia with central nervous system bleeding.

## Introduction

Chronic myeloid leukemia is a heterogeneous disease. Clinical course is categorized in three phases (Chronic, accelerated and blast crisis) with specific diagnostic criteria. Despite the definition of the phases, clinical presentation might be different, regarding the prognostic score, different response to the tyrosine kinase inhibitors and self features of each patient, furthermore of genetic variations that confer resistance to certain medications.

Lack of compliance of therapeutic regimen with tyrosine kinase inhibitors may lead to a blast crisis progression with a very bad prognosis. Nevertheless, a blast crisis may be a devastating scenario in a disease that has been considered cured with the new strategy approach.

Blast cells crisis in chronic myeloid leukemia comprises, in most cases, the myeloid or lymphoid phenotype. Myelomastocytic blast cell crisis is rare and the prognosis is not clearly defined. The objective of this case report is to show the clinical behavior of a patient treated at a reference hemato-oncology center in Colombia. This work has been published as an abstract (https://www.clinical-lymphoma-myeloma-leukemia.com/article/S2152-2650(18)30846-2/fulltext).

## Case presentation

We present the clinical case of a 29-year-old male patient treated at the Instituto Nacional de Cancerología of Colombia with a diagnosis of chronic myelogenous leukemia (CML) in high-risk chronic phase since February 2010. He started treatment with imatinib at a dose of 400 mg, obtaining a hematological response in the second month but not achieving a cytogenetic response in the 18th month. At that time, the patient continued treatment in another institution. It was possible to elucidate that the patient had a change of his treatment to dasatinib in March 2013 with the previous verification of the lack of cytogenetic response documenting a level of BCR-ABL transcription of 6.3%, period after which the patient, unfortunately, lasted eight months without treatment due to assurance problems. In September 2016, a BCR-ABL transcription level of 58% was documented, without a real knowledge about how much time he had taken dasatinib continuously at the moment of BCR/ABL evaluation; then, nilotinib treatment was begun.

**Figure 1:**
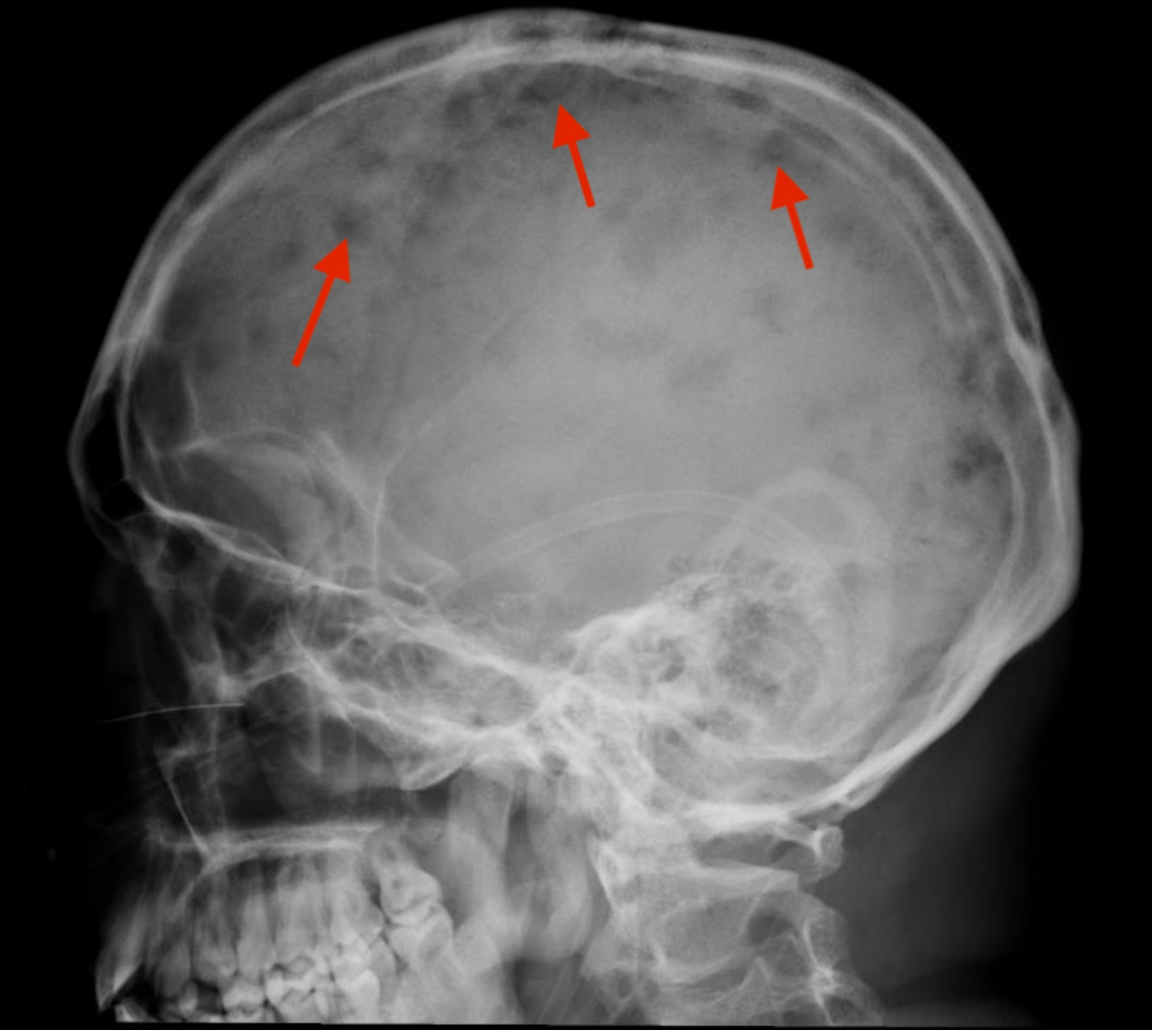
Multiple cranial lytic lesions.

The patient was readmitted to our institution in March 2017 and we diagnosed a progression to blast crisis of myeloid origin with a bone marrow study that documented 72% of blasts with karyotype without the growth of metaphases, being also very striking, the concomitant infiltrative cutaneous involvement, bone lesions of lytic type and hypercalcemia that required the use of zoledronic acid as an oncological emergency (Figure 1).

**Figure 2:**
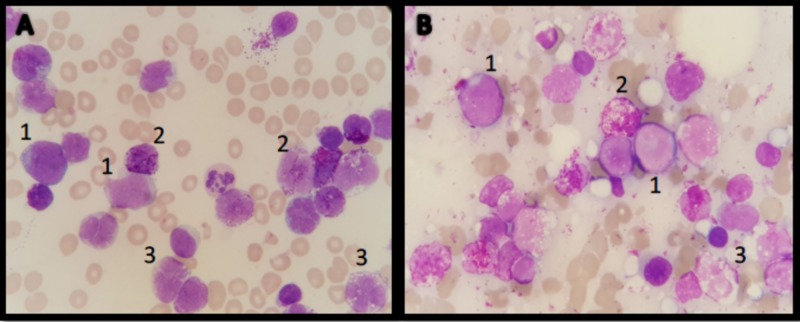
Cytomorphological examination, Wright 100x. (A) Peripheral blood and (B) bone marrow aspirate: 1. Blasts. 2. Multilobulated cells with abundant metachromatic granules and 3. Cells with lax chromatin, multi-loculated, basophilic hypogranular cytoplasm and/or with vacuoles.

**Figure 3:**
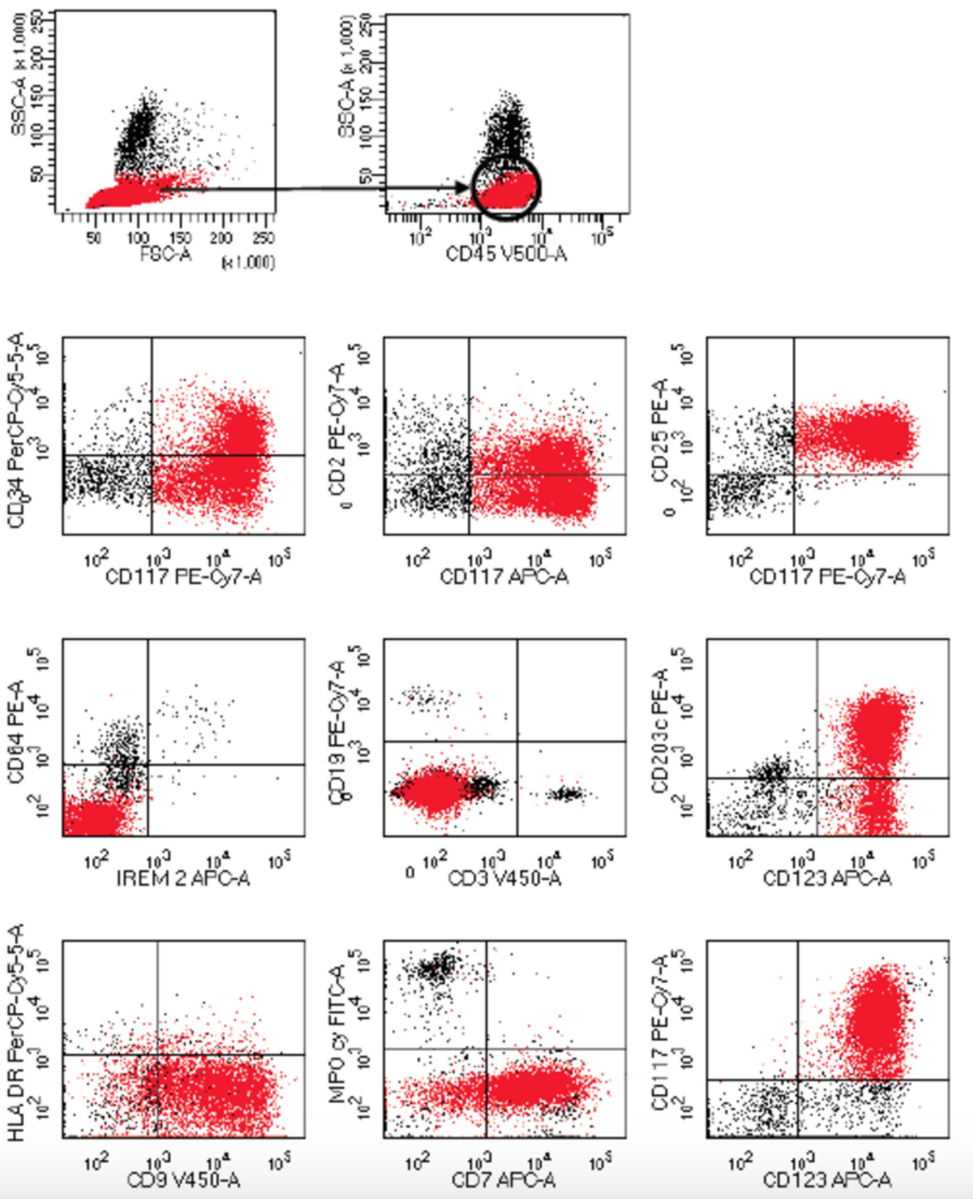
The flow cytometry study in bone marrow aspiration. It reveals a population of large size and complexity (red events) with intermediate CD45 and positivity for CD117, CD25, CD123 and CD9 heterogeneous with partial expression of CD34, CD2, CD203c and CD7. Note the negativity for MPO, CD64, IREM2, CD19, CD3s and HLA DR. This immunological profile has similarity between basophils vs mast cells, being more likely a mastocytic differentiation due to the strong expression of CD117, CD123 and CD25, interpreting these results as a chronic myeloid leukemia (CML) in blast crisis probably with mast cell differentiation.

At the end of the induction with 7 + 3 (seven days of cytarabine and three days of idarubicin) chemotherapy associated with bosutinib for 14 days and after several infectious complications, including invasive fungal infection and bacteremia due to *Enterococcus faecium*, as well as symptomatic hypocalcemia because of bisphosphonates, it was documented a percentage of blasts by flow cytometry of 29% in bone marrow and the existence of 46% of cells with basophilic versus mast cell characteristics on day 28 at the end of induction (Figures 2, 3).

**Figure 4:**
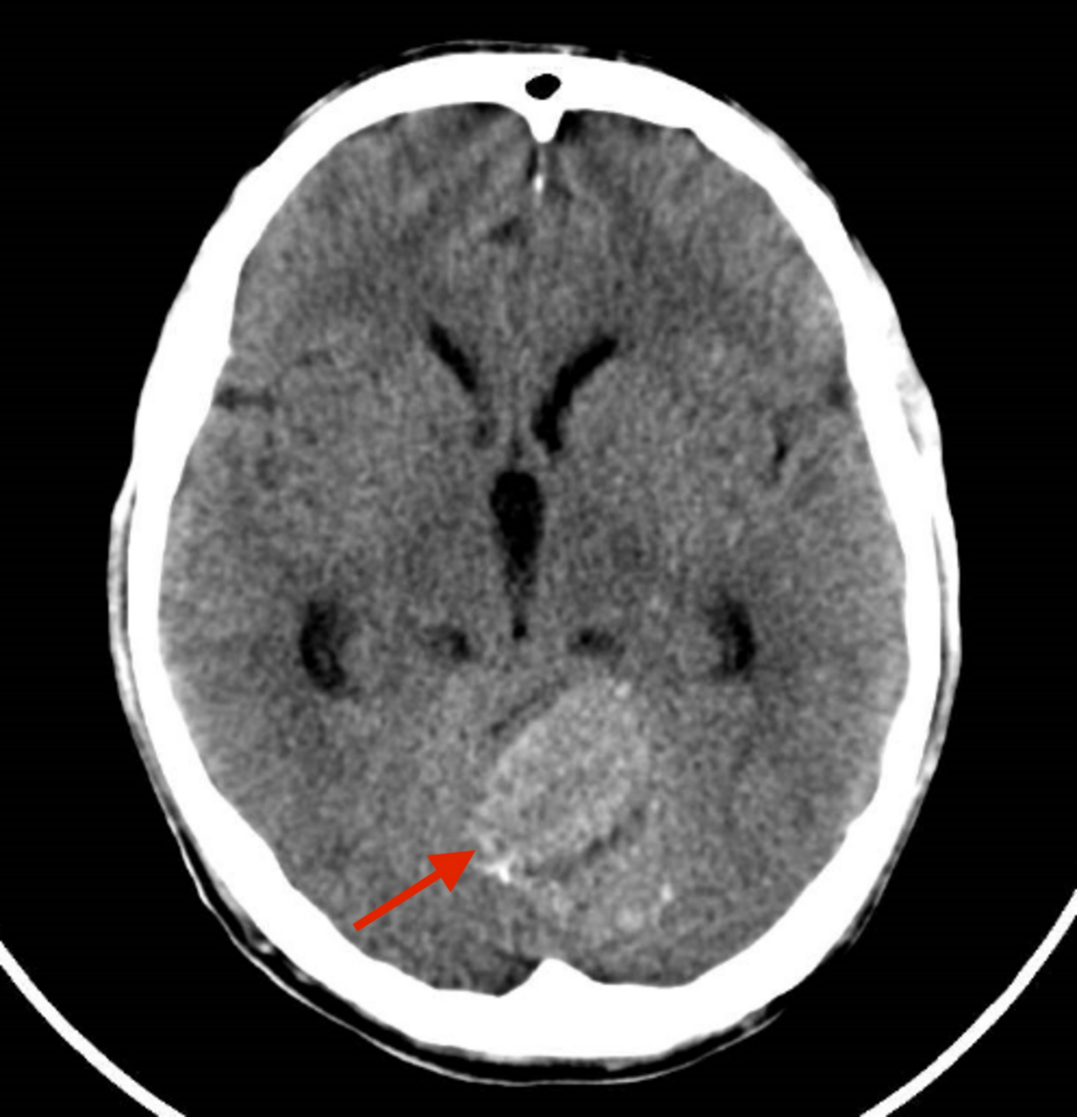
Computed tomography showing bleeding of the central nervous system.

A basophilic transformation was suspected versus aggressive systemic mastocytosis with a clonal, non-mastocytic hematological disorder (Figures 2, 3). Levels of serum tryptase and mutation D816V C KIT were requested, which were not reported. Treatment with CLAG-M (Cladribine, Cytarabine [Arabinosylcytosine-araC], granulocyte colony-stimulating factor [G-CSF], Mitoxantrone) was proposed, however, the patient died early in hyperleukocytosis and severe thrombocytopenia with central nervous system bleeding (Figure 4).

## Discussion

The CML has a three-phase behavior in which the clinical presentation of each phase, prognosis, and treatment are substantially different; the phases are: chronic phase (FC), accelerated phase (AF) and blast crisis (BC) [1]. In the past, virtually all patients progressed to BC with a median survival so short that they were usually measured in months. Despite the advent of current tyrosine kinase inhibitors (TKI) and the use of allogeneic hematopoietic stem cell transplantation, there is no significant improvement of overall survival [2]. The treatment of the disease in this phase is a challenge due to the high rates of therapeutic failure and mortality. The therapeutic schemes currently used are TKI associated with high-intensity systemic chemotherapy and allogeneic stem cell transplantation [2-4]. The choice of chemotherapy depends on the cellular phenotype of the acute leukemic transformation. The most common phenotype of BC is the myeloid phenotype comprising approximately two-thirds of the cases, followed by the lymphoid, megakaryocytic, erythroid, basophilic, and rarely monocytic and eosinophilic phenotype [5]. The BC with mastocytic differentiation is an absolutely rare entity and only two cases have been reported in the literature around the world [6,7]. The diagnosis is usually very difficult due to similar morphological, phenotypical and functional characteristics between basophilic blast crisis and mast cells, but the latter originate from a different precursor in the bone marrow. In addition, classifying the disease clinically is a challenge due to the characteristics shared with systemic mastocytosis. In our case, we did not have available tryptase levels or D816V C KIT mutation [7-10].

We present a patient diagnosed with CML, with a lack of compliance and/or resistant to the three tyrosine kinase inhibitors available for initial treatment, who progresses to blast cell crisis and in whose tests we demonstrate the existence of a population with mast cell differentiation that was difficult to differentiate from the blast cell population. After a rescue plan using chemotherapy for myeloid blast crisis associated with bosutinib, the patient died of central nervous system bleeding, and it was impossible to go ahead with a scheme based on cladribine as a second rescue. We have previously reported on the possibility of central nervous system bleeding in patients at an advanced stage of the disease [11].

## Conclusions

There are no guidelines on treatment in this scenario due to the rarity of this entity and it is assumed that patients with this clinical presentation have a high rate of mortality after they are diagnosed. Records of unusual BC presentations are necessary to define treatment strategies even if they are supported in retrospective studies.
